# The Mitogen-Activated Protein Kinase Kinase VdPbs2 of *Verticillium dahliae* Regulates Microsclerotia Formation, Stress Response, and Plant Infection

**DOI:** 10.3389/fmicb.2016.01532

**Published:** 2016-09-27

**Authors:** Longyan Tian, Yonglin Wang, Jun Yu, Dianguang Xiong, Hengjun Zhao, Chengming Tian

**Affiliations:** The Key Laboratory for Silviculture and Conservation of Ministry of Education, College of Forestry, Beijing Forestry UniversityBeijing, China

**Keywords:** *Verticillium dahliae*, MAP kinase pathway, microsclerotia formation, stress responses, pathogenicity

## Abstract

*Verticillium dahliae*, a ubiquitous phytopathogenic fungus, forms resting structures, known as microsclerotia that play crucial roles in Verticillium wilt diseases. VdHog1, a mitogen-activated protein kinase (MAPK), controls microsclerotia formation, virulence, and stress response in *V. dahliae*. In this study, we present detailed evidence that the conserved upstream component of VdHog1, VdPbs2, is a key regulator of microsclerotia formation, oxidative stress and fungicide response and plant virulence in *V. dahliae*. We identified VdPbs2, homologous to the yeast MAPK kinase Pbs2. Similar to the *VdHog1* deletion mutant, *VdPbs2* deletion strains exhibited delayed melanin synthesis and reduced formation of microsclerotia. When exposed to stresses, *VdPbs2* mutants were more sensitive than the wild type to osmotic agents and peroxide, but more resistant to inhibitors of cell wall synthesis and some fungicides. Finally, *VdPbs2* deletion mutants exhibited reduced virulence on smoke tree and tobacco seedlings. When taken together, we implicate that VdPbs2 and VdHog1 function in a cascade that regulates microsclerotia formation and virulence, but not all VdHog1 dependent functions are VdPbs2 regulated. This study thus provides novel insights into the signal transduction mechanisms that regulate microsclerotia formation and pathogenesis in this fungus.

## Introduction

The mitogen-activated protein kinase (MAPK) signaling pathways are involved in integrating multiple extracellular and intracellular signals to regulate transcription of specific genes that help the cell adapt to the conditions in eukaryotic cells (Gustin et al., 1998; Widmann et al., 1999). MAPK cascades consist of MAPK kinase kinases (MEKK or MAPKKK), MAPK kinases (MEK or MAPKK), and MAPK. The MAPK is activated by MEK, which is activated in turn by MEK kinase (Widmann et al., 1999). Activated MAPKs can then phosphorylate downstream substrates, affecting their biochemical properties and leading to specific output responses (Hamel et al., 2012). In *Saccharomyces cerevisiae* five MAPK pathways work in coordination, and in some cases independently, to regulate mating, invasive growth, cell wall integrity, ascospore formation and hyperosmoregulation (Gustin et al., 1998; Levin, 2005; Roman et al., 2007; Zhao et al., 2007).

Upon stress (osmotic, oxidative, acid and heat, etc), the high osmolarity glycerol (HOG) pathway is activated and the stress-activated MAPK Hog1 is phosphorylated (Brewster and Gustin, 2014). This pathway is initiated by two upstream branches, Sln1 and Sho1, and they converge at the Pbs2 MAPKK and are able to activate Pbs2, which then phosphorylates the MAPK Hog1 (Brewster et al., 1993; O’Rourke and Herskowitz, 2004; Roman et al., 2007). The activated Hog1 translocates into the nucleus and then regulates gene expression through several transcription factors, Hot1, Sko1,Smp1, Msn2, and Msn4 (Estruch and Carlson, 1993; Schüller et al., 1994; Gorner et al., 1998; Proft and Serrano, 1999; Rep et al., 2000; de Nadal et al., 2003). In particular, HOG pathway plays an important and somewhat specialized role in sensing stress conditions and activating gene expression, enabling the cell to resist the toxic effects of stress, survive and ultimately grow under adverse conditions (Gustin et al., 1998; Widmann et al., 1999).

Hog1 and its homologs in filamentous fungi are referred to as stress-activated MAPKs. Besides osmoregulation, homologs of Hog1 in pathogenic fungi are involved in pathogenesis and response to various stresses (Xu, 2000; Zhao et al., 2007; Hamel et al., 2012). In *Mycosphaerella graminicola*, strains lacking Hog1 homolog are impaired in pathogenicity (Mehrabi et al., 2006). In *Botrytis cinerea, SAK1* (Hog1 homolog) deletion mutants are unable to penetrate plant tissues (Segmuller et al., 2007). In oomycete *Phytophthora sojae*, silencing-mutants fail to colonize soybean (Li et al., 2010). However, some Hog1 homologs are dispensable for virulence, including *Magnaporthe oryzae OSM1* (Dixon et al., 1999), *Bipolaris oryzae SRM1* (Moriwaki et al., 2006), and *Colletotrichum orbiculare Osc1* (Kojima et al., 2004). In several fungal species, it has also been reported that HOG pathway contributes to resistance to a variety of fungicides (Zhang et al., 2002; Kojima et al., 2004). Pbs2, as the specific activator of Hog1, affects the response to hyperosmotic stress (Posas and Saito, 1997). Similar to Hog1, Pbs2 has proved to be involved in multiple stress responses in *S. cerevisiae* (Akhtar et al., 1997; Lai et al., 1997; Gustin et al., 1998). Furthermore, the Pbs2-Hog1 module controls stress response, differentiation and virulence in pathogenic fungi. For example, in *Cryptococcus neoformans, Candida albicans*, and *Cryphonectria parasitica, Pbs2* deletion mutants are hypersensitive to osmotic shock, high temperature, oxidative stress, and the antifungal drug fludioxonil, and attenuated in virulence (Arana et al., 2005; Bahn et al., 2005; Moretti et al., 2014).

*Verticillium dahliae*, a soil-borne plant pathogenic fungus, is responsible for Verticillium wilt diseases in more than 200 dicotyledonous plant species worldwide (Klosterman et al., 2011; Klimes et al., 2015). Notably, the microsclerotia with melanized particles in the interhyphal spaces confer resistance to UV irradiation, temperature extremes, enzymatic lysis, and fungicidal activities of the host plant (Gordee and Porter, 1961; Griffiths, 1970; Gessler et al., 2014). The high tolerance of microsclerotia allows the pathogen to survive under unfavorable conditions and prevents from chemical fungicides, and is thus an important aspect of pathogen fitness (Griffiths, 1970; Klosterman et al., 2009). Under optimal conditions, microsclerotia germinate to form hyphae in the soil, and penetrate the plant roots, where the fungus colonizes the xylem tissue of the plant vascular system. As disease progress, *V. dahliae* produces microsclerotia in dying plant tissues, which returned to the soil to initiate new primary infections. Because of their pivotal roles in pathogen survival and developmental processes, both of which linked to virulence, the microsclerotia are considered important targets for disease control (Gordee and Porter, 1961; Coley-Smith and Cooke, 1971; Duressa et al., 2013). Thus, elucidation of molecular mechanisms, especially the signal transduction pathways that regulate the development of microsclerotia, is essential for the development of novel control strategies.

Recently, dozens of genes that regulate microsclerotial development and virulence have been identified and functionally characterized in *V. dahliae*. Many of these genes are involved in MAP kinase signaling (*Msb, VMK1* and *Hog1*) (Rauyaree et al., 2005; Tian et al., 2014; Wang et al., 2016), cAMP-PKA-mediated signaling (*VdPKAC1*) (Tzima et al., 2010), G protein signaling (*VGB*) (Tzima et al., 2012), and other associated genetic networks (Duressa et al., 2013; Hu et al., 2014; Xiong et al., 2014; Klimes et al., 2015). Besides, transcription factors such as *VdCrz1* and *VdMcm1* were reported lately (Xiong et al., 2015, 2016). Although functional genomics of *V. dahliae* facilitates to uncover the molecular basis of microsclerotia formation, little else is known about the signal pathways involved in microsclerotia formation. Studies on genes of HOG pathway in *V. dahliae* have shown the essential role in expressing certain pathogenicity-related traits. Mutants lacking the transmembrane mucin Msb exhibit significant reductions in invasive growth, adhesive capacity, conidiation, and microsclerotia formation (Tian et al., 2014). In addition, our previous report showed that deletion of *VdHog1* delays microsclerotia formation, decreased virulence and heightened sensitivity to hyperosmotic stress (Wang et al., 2016).

In this study, we present evidence that *VdPbs2* regulates microsclerotia formation, stress responses and pathogenicity in *V. dahliae*. The *VdPbs2* deletion mutant exhibited delayed microsclerotia formation and reduced virulence on smoke tree and tobacco seedlings. Furthermore, deletion of *VdPbs2* also increased sensitivity to osmotic agents, while increasing resistance to some fungicides and compounds that interfered with cell wall synthesis. Taken together, these results indicate that the VdPbs2-VdHog1 module is important for microsclerotia formation, stress response and plant virulence in *V. dahliae*.

## Materials and Methods

### Fungal Strains and Growth Conditions

*Verticillium dahliae* wild type XS11 was isolated from a smoke tree, *Cotinus coggygria* in Fragrant Hills Park, Beijing (Wang et al., 2013). The spores of the wild type and its derivative mutants and its complementation strains were stored in 15% (v/v) glycerin at -80°C. To acquire conidia, all strains were activated and cultured on potato dextrose agar medium (PDA, containing 200 g of potato, 20 g of glucose, and 15 g of agar per liter) at 25°C and then collected after 7 days for generation of fresh hyphae, germination tests, and etc. For all stress assay, strains were cultured on solid complete medium (CM, 50 ml of 20× nitrate salts, 1 ml of 1000× trace elements, 10 g of glucose, 2 g of peptone, 1 g of yeast extract, 1 g of casamino acids, and 1 ml of vitamin solution per liter). To test sensitivity to osmotic stress, all strains were grown for 24 days on CM containing 0.8 M NaCl and 1.2 M sorbitol. For cell wall stress assay, all strains were grown on CM with 20 μg/ml Calcofluor White (CFW) (Sigma-Aldrich) and 50 μg/ml Congo Red (CR) (Sigma-Aldrich) for 3 and 7 days, respectively. For oxidative stress, agar diffusion tests were performed to measure the sensitivity of strains to H_2_O_2_, the same spore suspension (10^5^ spores/ml) of each strain were spread on PDA plates, and filter paper discs containing H_2_O_2_ (6, 12, and 18 mM) were placed in the center of each plate. The inhibition zone was determined after 3 days post inoculation (dpi). For fungicides assay, four different fungicides, such as 5 μg/ml difenoconazole (Sigma-Aldrich), 2 μg/ml chlorothalonil (Sigma-Aldrich), 10 μg/ml fludioxonil (Sigma-Aldrich), and 5 μg/ml iprodione (Sigma-Aldrich) were used. Three independent experiments of three replicates each were performed. To observe microsclerotia formation, conidia were sprayed onto the cellulose membrane (Ø = 80 mm; pore size = 0.22 μm) overlaid on solid basal medium (10 g of glucose, 0.2 g of sodium nitrate, 0.52 g of KCl, 0.52 g of MgSO_4_.7H_2_O, 1.52 g of KH_2_PO_4_, 3 μmol thiamine HCl, 0.1 μmol biotin, and 15 g of agar per liter). The microsclerotia formation were observed and photographed after incubation for every 48 h intervals. At 7 dpi, the observations were conducted every 7 days. All experiments were repeated at least three times.

### Bioinformatics Analysis

Information regarding *VdPbs2* was obtained from JGI^1^. Homologs of *VdPbs2* were identified using BLASTP searches of home databases of other fungal species (Broad Institute and Joint Genome Institute). Multiple sequence alignments were conducted using ClustalX 2.0 (Larkin et al., 2007). The phylogenetic tree was constructed using Mega6.0 (Tamura et al., 2013) with the Neighbor Joining algorithm under default settings and 1000 bootstrap replications.

### Targeted Disruption of *VdPbs2* and Mutant Complementation

To delete *VdPbs2* in the genome of *V. dahliae*, we used the split-marker method. First, the 1476 bp upstream (5′) and 1494 bp downstream (3′) flanking sequences of *VdPbs2* were amplified with primer pairs LY105/LY106 and LY107/LY108, respectively (**Supplementary Table S1**). The geneticin-resistance cassette was amplified with the Geneticinfor/Geneticinrev primers for deletion, which include approximately 20 bp that overlaps with the 5′ and 3′ flanking sequences, respectively. The two deletion cassettes resulting from fusion PCR with primer pairs LY105/Geneticinrev and Geneticinfor/LY108 (**Supplementary Table S1**) were used for protoplast transformation after sequencing. To obtain Δ*VdPbs2* complementation strains, the 3804 bp segment and the *VdPbs2*-GFP fusion construct were constructed containing the native promoter and coding region of *VdPbs2*. The 3804 bp segment for native complementation, amplified with primer pair LY109/LY166 (**Supplementary Table S1**) is used to restore the defects of Δ*VdPbs2* mutant. The *VdPbs2*-GFP fusion plasmid was constructed as follows. Firstly, a 3.76 kb genomic fragment was amplified with the primer pair LY105/LY167 (**Supplementary Table S1**), including the native promoter and the full *VdPbs2* open reading frame region. Then, it was inserted into the pKD5-GFP digested with *Sma*I. Confirmations were performed using PCR with the primer pairs LY137/LY165-RB, restriction digestion and sequencing. Finally, the native complementary segments of *VdPbs2* and *VdPbs2-GFP* fusion constructs were transformed along with a hygromycin-resistance cassette into Δ*VdPbs2* protoplasts using the PEG method (Wang et al., 2013). All transformants were verified using external screening primer pair LY137/LY138 and internal screening primers pair LY145/LY146 (**Supplementary Table S1**). The Δ*VdPbs2/Pbs2GFP* strain was preliminarily screened for GFP fluorescence and then verified using the external screening primer pair LY137/LY138 and the internal screening primer pair LY145/LY146 (**Supplementary Table S1**). Finally, southern blotting was performed to confirm the deletion of *VdPbs2* with the DIG High Prime DNA Labeling and Detection Starter Kit I in accordance with the manufacturers’ protocol (Roche, Germany). The genomic DNA of wild type and the deletion of *VdPbs2* strain was digested with *Kpn*I and hybridized with a probe amplified from the *V. dahliae* strain XS11 genomic DNA with LY170up/LY170down (**Supplementary Table S1**) and labeled with the DIG primer.

### RNA Extraction and Quantitative Real-Time PCR

Fresh mycelium of Δ*VdPbs2* mutants and wild type were cultured in CM at 25°C for 5 days and collected with single-layer miracloth. Mycelia were subjected to RNA extraction using TRIzol reagent (Invitrogen) and purified with the RNA Mini Kit (Ambion). RNA integrity was confirmed by agarose gel electrophoresis. Reverse-transcription PCR was performed with Oligo-DT and SuperScript III reverse transcriptase (Invitrogen). Quantitative real-time PCR (qRT-PCR) was performed with SuperReal Premix Plus (TIANGEN, China) on an ABI 7500 real-time PCR system (Applied Biosystems, USA). The *β-tubulin* of *V. dahliae* is used as an internal reference. Relative expression levels were calculated using the ΔΔCT method (Livak and Schmittgen, 2001). All primers used in this study are listed in **Supplementary Table S1**.

### Pathogenicity Assays

To test the ability of penetration of Δ*VdPbs2* mutant, spores were dropped onto onion epidermis at the concentration of 10^4^ conidia/ml. At 32 hpi, the penetration was observed after staining with aniline blue under light microscopy (DM2500, Leica). To determine the pathogenicity of the Δ*VdPbs2* mutant, spores were filtered from liquid CM after 10 days of cultivation and then diluted to 10^6^/ml with distilled water. One-year-old smoke tree seedlings were selected for inoculation and soaked in the conidia suspension for 10 min. The seedlings were then replanted in autoclaved soil and observed at regular intervals. To determine whether specific strains could invade the seedlings, the seedling stems were clipped into tiny fragments for isolation 14 days after inoculation (Xiong et al., 2015). Tobacco seedlings were also used for virulence tests using the same methods. The height of tobacco seedlings were measured at 30 dpi.

### Microscopic Observation and Localization of *VdPbs2*

To analyze the response to stress, mycelium of the wild type and *VdPbs2* deletion mutant were inoculated in the CM with 0.8 M NaCl for 4 days, then myceliua were collected for observation. Pictures were taken using the microscope (Leica DM 2500). To analyze of subcellular localization of *VdPbs2*, conidia and hyphae were collected from liquid CM. Then the fluorescence of mycelium and conidia treated with 0.8 M NaCl for 2 h were observed. The pictures were acquired using a Leica SP5 confocal laser-scanning microscope. A diode laser, Argon/2 (458, 477, 488, 496, 514 nm) was used, and the fluorescence filters were EX 488; EM 510/40. The quantification of image fluorescence was performed using the Adobe Photoshop software.

### Statistical Analysis

The melanized area fraction was measured using ImageJ^2^ under the default settings (all the threshold of image was 42.589) (Papadopulos et al., 2007). Data were expressed as mean values ± standard error of the mean. Statistical analyses were performed by using Student’s *t*-test. A *p*-value < 0.05 was considered as statistically significant.

## Results

### Generation of the *VdPbs2* Mutant

To investigate whether the other component of HOG signaling pathway affects the physiology and morphology of *V. dahliae*, we identified the homolog of *S. cerevisiae Pbs2* in the *V. dahliae* genome database. A gene encoding a MAPK kinase (VDAG_02783) was designated as *VdPbs2*. The protein contains two kinase motifs (residues 258–280 and 325–563) and a tyrosine kinase domain, Pkinase_Tyr (residues 322–559, marked with dashed lines in **Supplementary Figure S1**). Subsequent phylogenetic analysis and amino-acid sequence alignments revealed that *VdPbs2* has high sequence similarity with *Pbs2* homologs in other fungi, particularly those in *V. alfalfae* and *N. crassa*. Moreover, RNA-Seq revealed that expression levels of *VdPbs2* increase during microsclerotial development at 60 h, 72 h, 96 h, and 14 days in XS11 strain (Xiong et al., 2014).

Two deletion mutants (Δ*VdPbs2*-22 and Δ*VdPbs2*-32) were verified by PCR and Southern blots (**Supplementary Figure S2**). The complemented strain Δ*VdPbs2/Pbs2GFP* was confirmed to harbor the full-length *VdPbs2* gene (**Supplementary Figure S2**) and restore phenotypes of the Δ*VdPbs2* mutant (**Supplementary Figure S3**). The results showed that the deletion mutants and complementation strain (Δ*VdPbs2/Pbs2* and Δ*VdPbs2/Pbs2GFP*) were successfully generated.

### *VdPbs2* is Involved in Microsclerotia Formation and Melanin Biosynthesis

**FIGURE 1:**
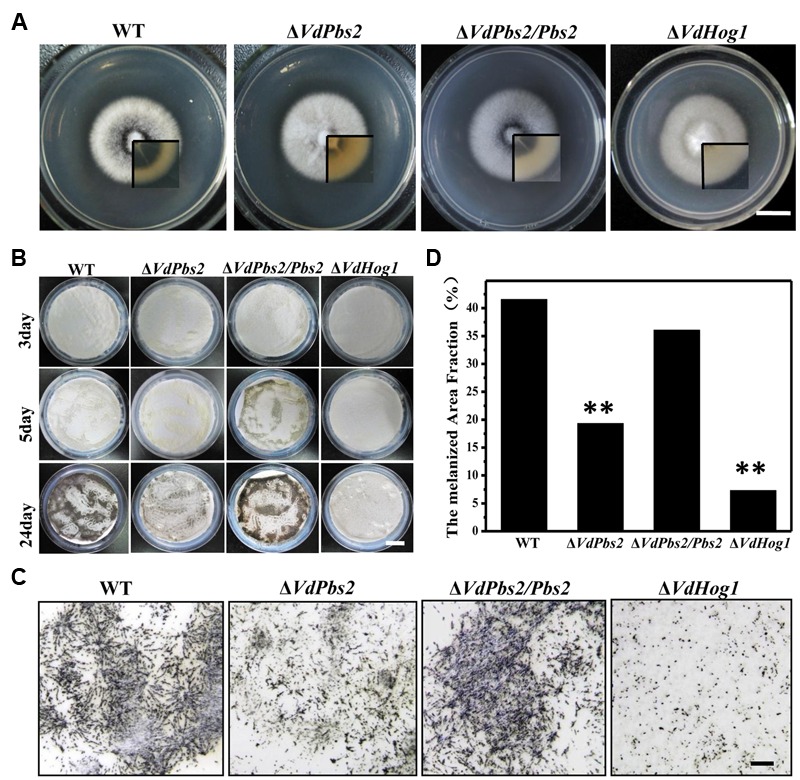
**Loss of *VdPbs2* leads to reduced microsclerotia formation. (A)** Colony morphology of the wild type, Δ*VdPbs2*, Δ*VdPbs2/Pbs2* and Δ*VdHog1* grown on PDA for 8 days. The inset shows colony from the opposite view. **(B)** Microsclerotia formation of the individual strain on cellulose membrane placed onto basal medium plates, and incubated at 25°C at 3, 5, and 24 days. Conidia from each strain were sprayed on the cellulose membrane at a concentration of 10^5^ conidia/ml. **(C)** Microscopic observation of microsclerotia formation of the above four strains at 24 dpi. Scale bar = 1 mm. **(D)** Melanized area fractions in the colony were counted by ImageJ. Asterisk indicates significant difference at *P* < 0.01.

To investigate the role of *VdPbs2* in microsclerotia formation, we first paid our attention to the connection between *VdPbs2* function and axenic growth on plate media. Similar to the Δ*VdHog1* mutant, Δ*VdPbs2* mutants exhibited no significant difference in growth rate but delayed to form microsclerotia on PDA compared with the wild type (**Figure 1A**). Few melanized microsclerotia can form in the Δ*VdPbs2* mutant; by contrast, abundant melanized microsclerotia were produced in the wild type and the Δ*VdPbs2/Pbs2* strain (**Figure 1A**). To determine the influence of *VdPbs2* on microsclerotia in detail, we observed the microsclerotia formation on BM. The wild type and the Δ*VdPbs2/Pbs2* strain started to accumulate a small amount of melanized microsclerotia at 3 dpi, however, a small number of melanized microsclerotia were observed in the Δ*VdPbs2* mutant at 7 dpi (**Figure 1B**). Furthermore, at 24 dpi, the Δ*VdPbs2* and Δ*VdHog1* strains still had significant defects in microsclerotia formation, and the melanized area fraction of each strain revealed the deficiency in the melanin accumulation in Δ*VdPbs2* and Δ*VdHog1* mutants when compared with wild type and the Δ*VdPbs2/Pbs2* strain (**Figures 1C,D**). Strikingly, the melanized microsclerotia were significantly less in the Δ*VdHog1* mutant than that of in the Δ*VdPbs2* mutant (**Figure 1**), indicating that *VdHog1* may play a more prominent role in the formation of melanized microsclerotia.

**FIGURE 2:**
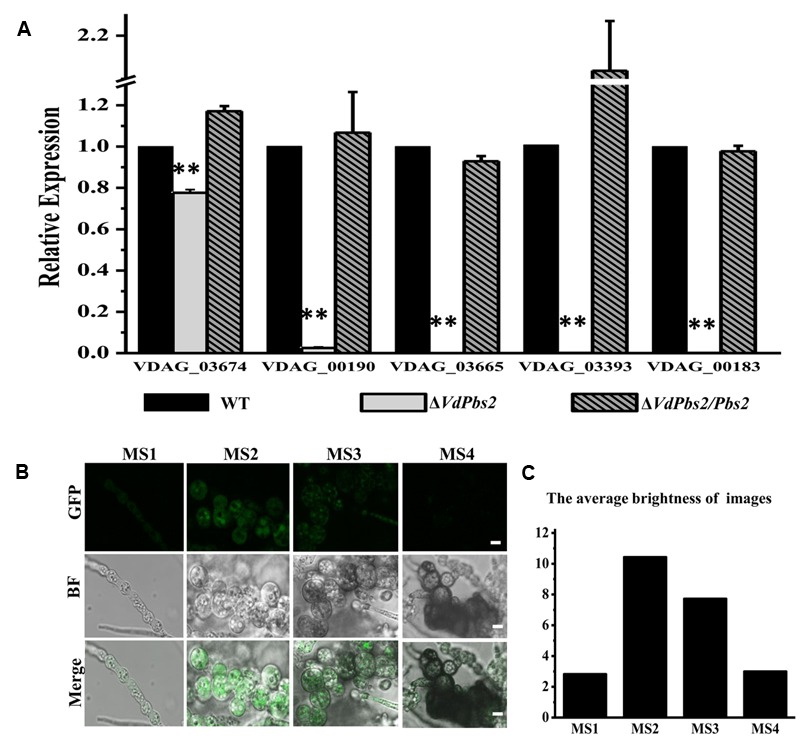
**The expression of genes involved in melanin biosynthesis in the *VdPbs2* mutant. (A)** Expression of five melanin related genes (VDAG_03674, VDAG_00190, VDAG_03665, VDAG_03393 and VDAG_00183) during microsclerotia formation. The *β-tubulin* was used as an internal reference gene. Total RNA was directly extracted from mycelium of the wild type, Δ*VdPbs2*, and Δ*VdPbs2/Pbs2* grown on PDA plates for 8 days. Error bar represents standard deviation. Asterisk indicates significant difference at *P* < 0.01. **(B)** Expression patterns of *VdPbs2-GFP* during microsclerotia development. GFP expression driven by the native promoter of *VdPbs* was examined using fluorescence microscope. Spores were cultivated in CM liquid for 4 days. MS1-MS4 represents four typical stages during the entire process of microsclerotia formation at 60 (mycelium at the early stage of inflation), 72 (mycelium inflated completely but without melanin accumulation), 96 h (inflated mycelium with the slight accumulation of melanin), and 14 days (inflated mycelium with the massive accumulation of melanin). Scale bar = 10 μm. **(C)** The quantification of images fluorescence correlated with **Figure 3B**. The average brightness of image was performed using the Adobe Photoshop software.

Consistent with reduced melanin accumulation in the Δ*VdPbs2* mutant, genes associated with melanin synthesis were expressed at significantly lower levels in Δ*VdPbs2* mutant (**Figure 2A**). Notably, of five melanin-related genes, four genes (VDAG_00190, VDAG_03665, VDAG_03393, and VDAG_00183) were more than 50-fold down-regulated in Δ*VdPbs2* mutant compared with the wild type and the Δ*VdPbs2/Pbs2* complementation strain (**Figure 2A**). The result was consistent with expression profiles of these genes in Δ*VdHog1* mutant (Wang et al., 2016). Furthermore, we tested the expression analysis and subcellular localization of *VdPbs2* fused with GFP under the control native promoter of *VdPbs2*. The results demonstrated that *VdPbs2* was significantly upregulated during microsclerotia formation and green fluorescence remained a higher level at the early stage of microsclerotia formation (**Figures 2B,C**). Taken together, these observations indicate *VdPbs2* is required for melanized microsclerotia formation via the Hog1-mediated pathway.

### Deletion of *VdPbs2* Impairs Fungal Growth under Osmotic Stress Conditions

**FIGURE 3:**
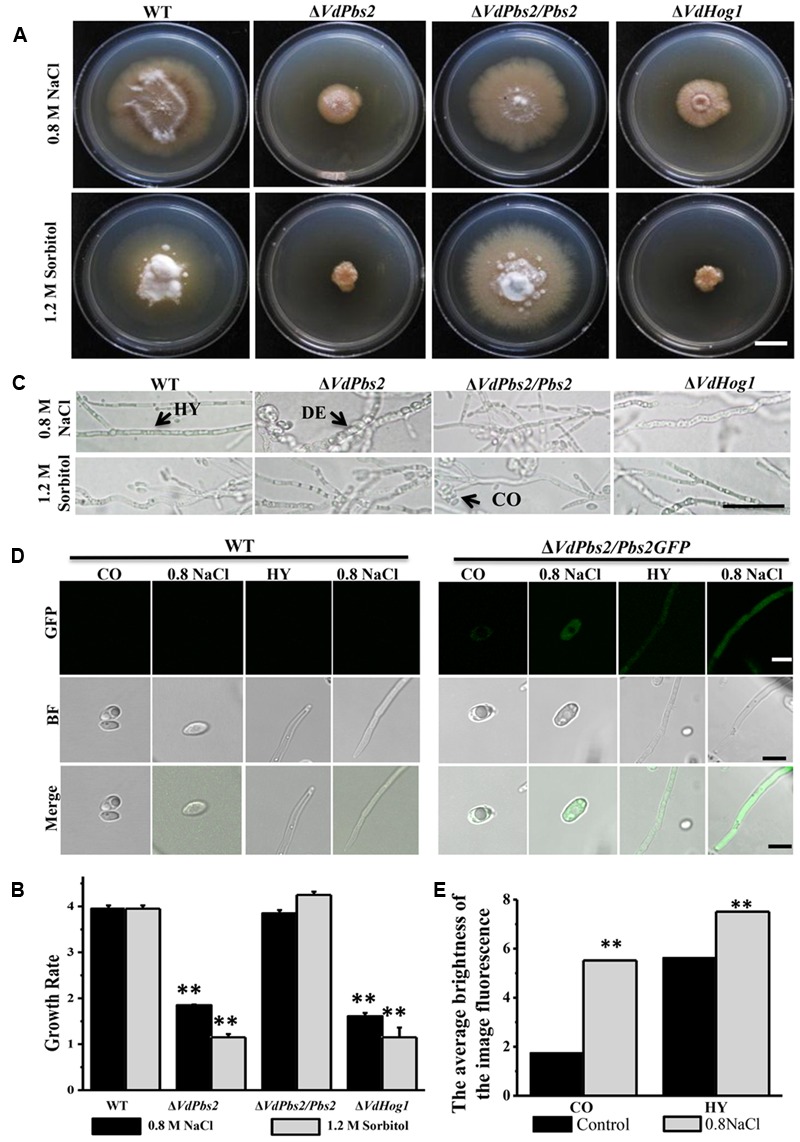
**Deletion of *VdPbs2* impairs fungal growth under osmotic stress with hyphal lysis. (A)** Colony morphology of the wild type, Δ*VdPbs2*, Δ*VdPbs2/Pbs2*, and Δ*VdHog1* grown at 25°C for 20 days on CM containing 0.8 M NaCl and 1.2 M sorbitol, respectively. Scale bar = 1 cm. **(B)** The growth rate of the individual strain on CM under osmotic agents. All assays were performed in triplicate. Error bars represent standard deviations. Asterisk indicates significant difference at *P* < 0.01. **(C)** Hyphal morphology of the four above strains treated by 0.8 M NaCl and 1.2 M sorbitol, respectively. Under hyperosmotic conditions, the mycelium of the mutant was deformed. HY = hyphae, CO = conidia, DE = deformity. Scale bar = 10 μm. **(D)** Expression pattern of *VdPbs2-GFP* in response to osmotic stress at conidia and hyphae. The conidia and hyphae of Δ*VdPbs2/Pbs2GFP* strains were treated with 0.8 M NaCl for 2 h compared with that of the wild type. HY = hyphae, CO = conidia. Scale bar = 5 μm. **(E)** The quantification of images fluorescence correlated with **(D)** in the Δ*VdPbs2/Pbs2GFP* strain.

To investigate the function of *VdPbs2* in the response to hyperosmotic stress, strains were grown on CM supplemented with 0.8 M NaCl and 1.2 M sorbitol, respectively. When grown on minimal media containing 0.8 M NaCl and 1.2 M sorbitol, respectively, Δ*VdPbs2* mutant, compared to the wild type and the Δ*VdPbs2/Pbs2* strain was dramatically reduced for growth, which was similar to Δ*VdHog1* mutant (**Figures 3A,B**). Besides, clear hyphal lysis occurred in both Δ*VdPbs2* and Δ*VdHog1* mutants indicated by hyphae deformities visible on the above media (**Figure 3C**). As shown in **Figures 3D,E**, cytoplasmic distribution of VdPbs2 was clearly observed after treated with 0.8 M NaCl. Collectively, the results suggested that VdPbs2-VdHog1 module contributes to the response to osmotic stress in *V. dahliae*.

### Loss of *VdPbs2* Increases Resistance to Cell Wall Stress

**FIGURE 4:**
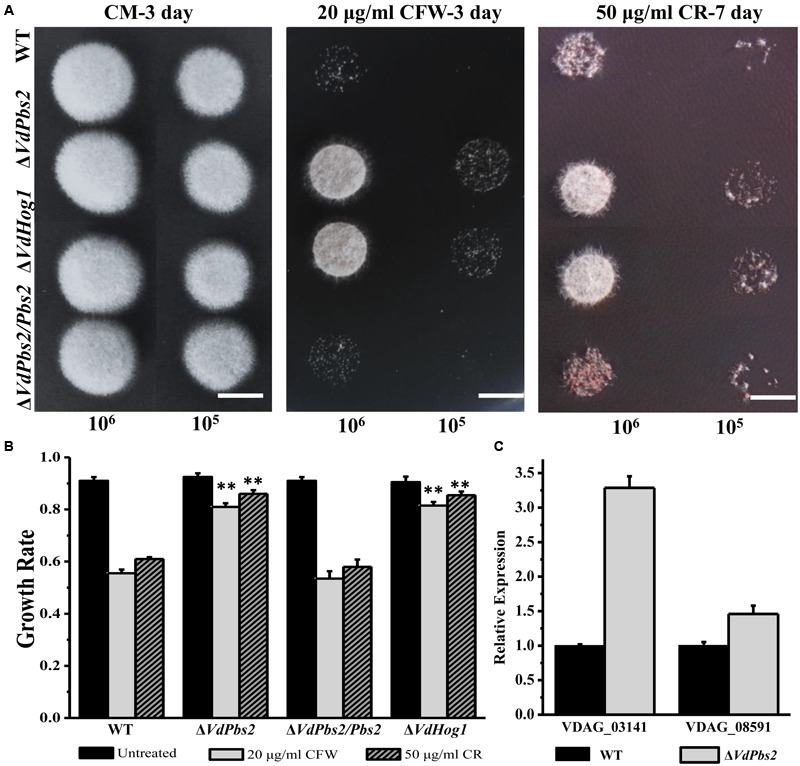
**Loss of *VdPbs2* increases resistance to cell wall stress. (A)** Stress responses of wild type, Δ*VdPbs2*, Δ*VdPbs2/Pbs2*, and Δ*VdHog1* strains on CM containing 20 μg/ml CFW and 50 μg/ml CR, respectively. Images were taken at 3 dpi for CFW and 7 dpi for CR. In all assays, the plates were inoculated with conidial solution of wild type, Δ*VdPbs2*, Δ*VdPbs2/Pbs2*, and Δ*VdHog1* strains. Conidial suspension (10^5^/ml and 10^6^ /ml) of the individual strain were spotted on CM media containing the indicated concentration CFW and CR, Scale bar = 0.5 cm. **(B)** Relative growth of wild type, Δ*VdPbs2*, Δ*VdPbs2/Pbs2*, and Δ*VdHog1* strains treated by the indicated cell stress. Error bar represents standard deviation. Asterisk indicates significant difference at *P* < 0.01. **(C)** The expression of two genes (VDAG_08591 and VDAG_03141) involved in chitin synthesis was increased in the Δ*VdPbs2* mutant. Error bars indicate standard deviations derived from three independent experiments consisting of three replicas each.

To determine whether deletion of *VdPbs2* affects the response to cell wall stress in *V. dahliae*, we tested cell viability of the Δ*VdPbs2* mutant under cell wall stressors such as CFW and CR. Conidia (10^5^ conidia/ml and 10^6^ conidia/ml) of Δ*VdPbs2*, Δ*VdHog1*, wild type, and Δ*VdPbs2/Pbs2* strain were spotted on CM media containing CFW (20 μg/ml) and CR (50 μg/ml), respectively. Enhanced growth on media with CFW (20 μg/ml) and CR (50 μg/ml), respectively, was observed for Δ*VdPbs2* and Δ*VdHog1* mutants. By contrast, reduced growth was observed for the wild type and Δ*VdPbs2/Pbs2* strain (**Figures 4A,B**), suggesting *VdPbs2* and *VdHog1* are involved in the response to cell wall stress.

We next sought more evidence for a functional connection between *VdPbs2* and cell wall assembly. To determine the expression profiles of genes encoding chitin synthase, we used qPCR to analyze RNA extracted from wild type and Δ*VdPbs2* mutant strains grown in liquid shake CM for 5 days. Loss of *VdPbs2* function induced the expression of chitin synthase genes (VDAG_08591 and VDAG_03141) compared to wild type (**Figure 4C**). Thus, genes for chitin synthase are misregulated in Δ*VdPbs2* mutant when compared with wild type, accounting for enhanced resistance to cell wall stressors. Summarily, these results demonstrate that *VdPbs2* may negatively regulate cell wall synthesis.

### *VdPbs2* is Essential for the Oxidative Stress Response

**FIGURE 5:**
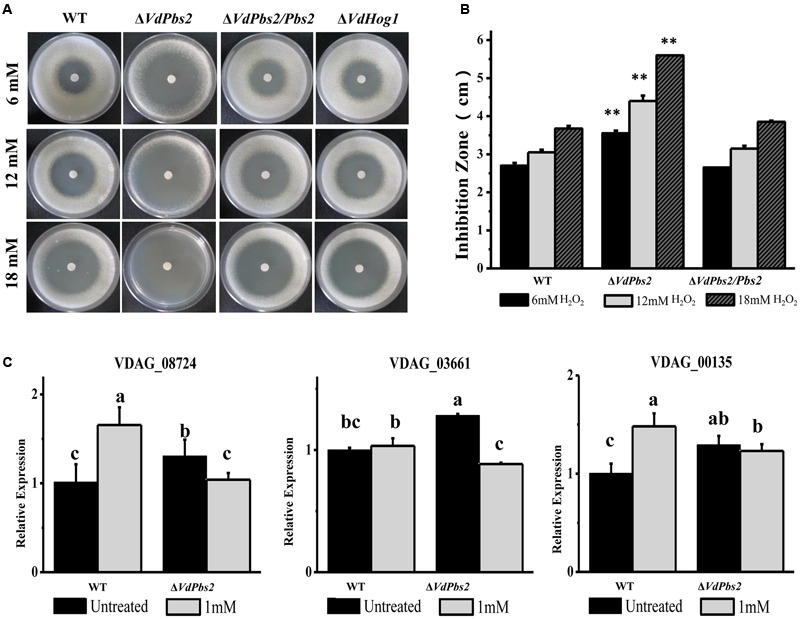
***VdPbs2* contributes to the oxidative stress response. (A)** The Δ*VdPbs2* and Δ*VdHog1* mutants were compared with the wild type and the Δ*VdPbs2/Pbs2* strain. Equal conidial suspension (10^5^ spores/ml) of each strain was sprayed on PDA plates. Sterile filter paper disks with 5 mm diameters were placed in the center of the plates, and 10 μL of 6, 12, and 18 mM H_2_O_2_ were added to each paper disk, respectively. The plates were incubated at 28°C for 4 days and the inhibition zones were measured Scale bar = 1 cm. **(B)** Zones of growth inhibition in **(A)** were quantified. Error bars represent standard deviation. Asterisk indicates significant difference at *P* < 0.01. **(C)** Downregulation of genes related to peroxidase in the Δ*VdPbs2* mutant. Relative expression levels of three genes (VDAG_08724, VDAG_03661 and VDAG_06340), which encode peroxidases, were determined by qRT-PCR using the RNA from mycelium treated with 1 mM H_2_O_2_ for 30 min. Error bars represent standard deviation.

To evaluate the responses of the Δ*VdPbs2* mutant to oxidative stress, the inhibition zone was measured on the media containing H_2_O_2_. As shown in **Figures 5A,B**, the Δ*VdPbs2* mutant exhibited the larger inhibition zones than the wild type and the Δ*VdPbs2/Pbs2* strain at a different concentration of H_2_O_2_ suggesting that *VdPbs2* is required for H_2_O_2_ detoxification. In addition, consistent with our previous observations, loss of *VdHog1* did not abolish oxidative sensitivity in *V. dahliae* (**Figure 5A**). Furthermore, based on sequence homology, we identified genes encoding H_2_O_2_ detoxification in *V. dahliae*. Transcript analysis revealed that three genes (VDAG_08724, VDAG_03661, and VDAG_06340) were consistently down-regulated in the Δ*VdPbs2* mutant compared to that of the wild type after treated with 1 mM H_2_O_2_ for 30 min (**Figure 5C**). Thus, *VdPbs2* is essential for the oxidative stress response, but not *VdHog1*.

### *VdPbs2* Deletion Mutants Exhibit Distinct Responses to Different Fungicides

**FIGURE 6:**
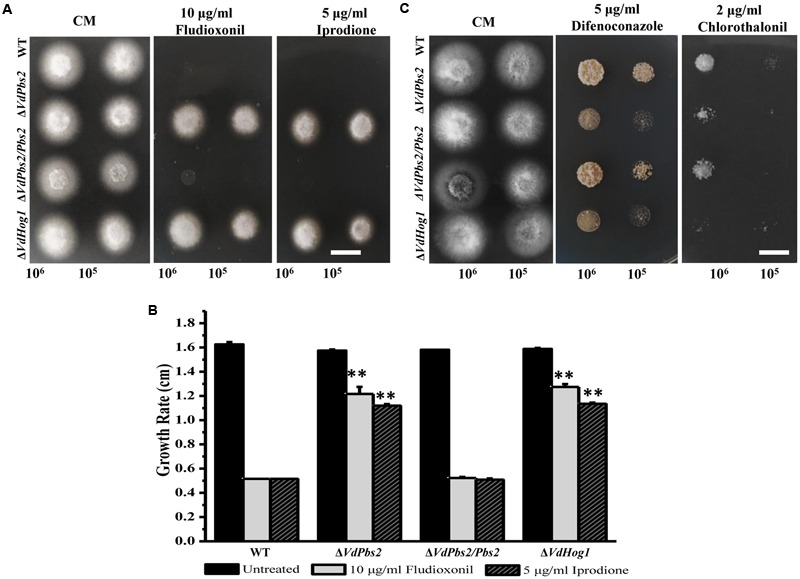
***VdPbs2* deletion mutants exhibit distinct responses to different fungicides. (A)** The Δ*VdPbs2* and Δ*VdHog1* mutants showed enhanced resistance to fludioxonil and iprodione. Conidial suspension (10^5^/ml and 10^6^ /ml) of the wild type, Δ*VdPbs2* and Δ*VdHog1*, and the the Δ*VdPbs2/Pbs2* were spotted on CM media with the indicated concentration of fludioxonil and iprodione, respectively. **(B)** The Δ*VdPbs2* and Δ*VdHog1* mutants exhibited more sensitivity to chlorothalonil and difenoconazole. Conidial suspension (10^5^/ml and 10^6^ /ml) of the above strains were spotted on CM media with the indicated concentration of chlorothalonil and difenoconazole, respectively. **(C)** Growth rate of the above strains on CM containing with the indicated concentration of fludioxonil and iprodione, respectively. Error bar represents standard deviation. Asterisk indicates significant difference at *P* < 0.01.

*VdHog1* deletion mutant is highly resistant to the fungicide fludioxonil (Wang et al., 2016). To determine if deletion of *VdPbs2* affects the response to fungicides, we tested the sensitivity of the Δ*VdPbs2* mutant to various fungicides. Similar to the response of the Δ*VdHog1* mutant to fungicides, the Δ*VdPbs2* mutant exhibited enhanced resistance to fludioxonil and iprodione and increased sensitivity to chlorothalonil and difenoconazole, respectively, when compared with the wild type and the Δ*VdPbs2/Pbs2* strain (**Figure 6**), suggesting that *VdPbs2* is involved in accumulation of osmoprotectant molecules of fungal cell in the response to fungicidal compounds.

### *VdPbs2* is Required for Plant Infection

**FIGURE 7:**
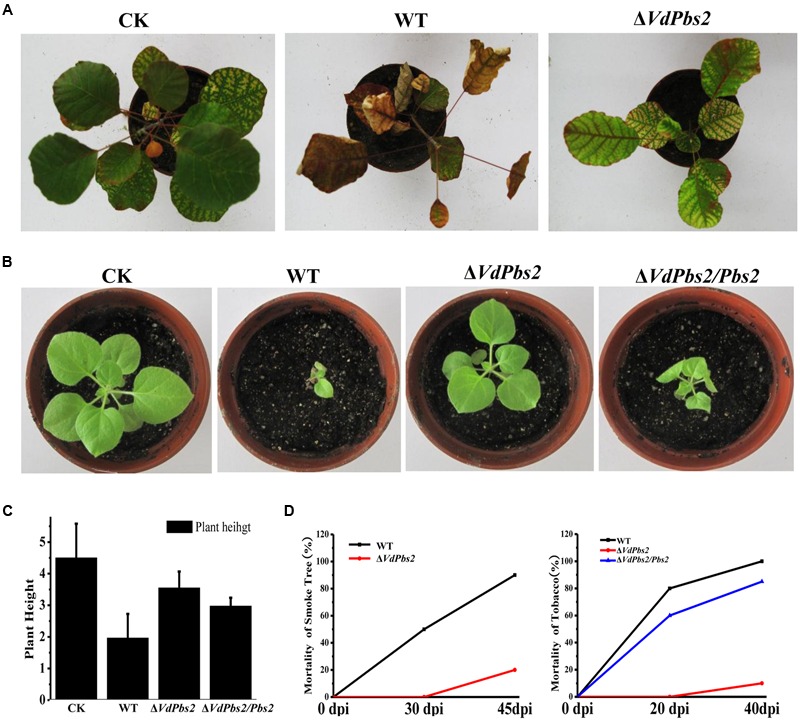
**Reduced virulence of the *VdPbs2* mutant on smoke tree and tobacco seedlings. (A)** One-year-old smoke trees were inoculated with conidia concentration of 10^6^/ml of the wild type and Δ*VdPbs2* mutant. The pictures were taken at 45 dpi. Twenty seedlings were inoculated with each strain. **(B)** Two-month-old tobacco seedlings were inoculated with the same methods mentioned in **(A)**. The assays were performed in triplicate. The pictures were taken at 40 dpi. **(C)** Height of tobacco seedlings inoculated with the above strains. The height of tobacco seedlings measured at 30 dpi. **(D)** The mortality of smoke tree (at 30, 45 dpi) and tobacco (at 20, 40 dpi) inoculated with the wild type, Δ*VdPbs2* and Δ*VdPbs2/Pbs2* strains.

We next sought to address whether *VdPbs2* plays a role in virulence in plants. We used seedlings of smoke tree and tobacco to carry out the virulence experiments. On both hosts, the Δ*VdPbs2* mutant exhibited striking reduced virulence (**Figures 7A,B**) and only less 20% mortality of plants at 45 dpi (**Figure 7D**). By contrast, at 45 dpi, up to 80% mortality of which inoculated with the wild type and the Δ*VdPbs2/Pbs2* strain showed clear wilt symptoms, including chlorosis (**Figures 7A,B**) and obviously reduced plant height (**Figures 7B,C**). Due to the limitations, we just further observed the penetration of the strain on onion epidermis. The wild type could infect epidermal cells and expand into the epidermal tissues, whereas the Δ*VdPbs2* mutant hardly infects epidermal cells even though the mutants produced long germ tubes (**Supplementary Figure S4**). Together, these results indicated that *VdPbs2* may be involved in the penetration process during plant infection.

## Discussion

In this study, we investigated the role of *VdPbs2* in the development of microsclerotia and pathogenicity in *V. dahliae*. Similar to the *VdHog1* deletion mutant, *VdPbs2* deletion mutants exhibited reduced microsclerotia formation, heightened sensitivity to osmotic stress, enhanced resistance to chemicals that interfered with cell wall synthesis and attenuated virulence on seedlings of smoke trees and tobacco. Strikingly, *VdPbs2* plays a crucial role in the response to oxidative stress, whereas *VdHog1* is dispensable for the response to oxidative stress. These results suggest that the module of VdPbs2-VdHog1 function in a signaling cascade that regulates stress response, developmental processes and pathogenicity in *V. dahliae*.

Microsclerotia with melanized particles in the interhyphal spaces confer resistance to adverse conditions (Gordee and Porter, 1961; Griffiths, 1970; Gessler et al., 2014). Genes involved in melanin biosynthesis in *V. dahliae* play crucial roles in the formation of fully functional microsclerotia (Griffiths, 1970; Wheeler et al., 1976; Xiong et al., 2014). In this study, *VdPbs2* mutants exhibited significantly reduced microsclerotia formation (**Figures 1A,B**). In addition, five genes involved in melanin biosynthesis were also significantly downregulated in the Δ*VdPbs2* mutant (**Figure 2A**). Although both *VdHog1* and *VdPbs2* were identified to positively regulate microsclerotia formation and melanin biosynthesis, *VdHog1* has a stronger influence on melanized microsclerotia (**Figures 1A–D**). Accordingly, we speculate that *VdHog1* possibly plays a more crucial role in the regulation of microsclerotia formation than *VdPbs2*. These findings emphasize that loss of Pbs2 and Hog1, the vital components of the HOG MAPK signal transduction pathway, delayed melanin synthesis and microsclerotia maturation in *V. dahliae*.

Mutants of *VdPbs2* and *VdHog1* also exhibited elevated sensitivity to osmotic stress, which identical with the other studies in yeast (Alonso-Monge et al., 2001), *F. proliferatum* (Adám et al., 2008), *C. albican*s (Alonso-Monge et al., 2009). Fungal cell wall mediates all signals between cells and their environment (Latge and Beauvais, 2014). Moreover, HOG signaling functionally participates in the maintenance of cell wall architecture (Garcia-Rodriguez et al., 2000) and *C. albicans* (Arana et al., 2005; Navarro-Garcia et al., 2005). In *S. cerevisiae*, there exist, two osmosensing signal transduction pathways, one is the HOG pathway and the other is the PKC-MAPK pathway, which respond to hypertonic and hypotonic shock, respectively (Davenport et al., 1995). Subsequently, some evidence revealed shared targets of the PKC1 pathway with high-osmolarity response routes (Alonso-Monge et al., 2001). The PKC-MAPK signaling pathway were reported to be vital to maintaining integrity of the cell and affected the location of cell wall components, the formation of melanin and responding to the osmotic and cell wall-inhibiting agents in pathogenic fungi (Davenport et al., 1995; Gerik et al., 2008), which indicated it is attractive targets for developing novel strategies to control pathogen. In *C. albicans*, Mkc1, the component of PKC-MAPK pathway, is phosphorylated under some stimuli and its function is partially dependent on the presence of Hog1 (Navarro-Garcia et al., 2005). Our study showed that both *VdPbs2* and *VdHog1* mutants exhibit altered susceptibility to CFW and CR, which inhibit fungal cell wall assembly by binding to chitin and β-1, 4-glucans, respectively. Furthermore, two genes related to chitin synthase were indeed significantly upregulated in the *VdPbs2* deletion mutant. Therefore, we inferred that *VdPbs2* and *VdHog1* negatively regulate cell wall synthesis, thereby affecting some proteins involved in the PKC1 cascade, the major signaling pathway responsible for sensing cell integrity, suggesting potential cross-talk between the Hog1 and Mpk1 MAPK pathways. It has been reported in some filamentous fungi that Mkk1 played a critical role in the crosstalk between the PKC and HOG regulatory pathways (Li et al., 2012; Zheng et al., 2012; Yin et al., 2016).

Regarding the oxidative stress response, the roles of Pbs2 and Hog1 are more complicated. In *S. cerevisiae*, the HOG pathway is required for oxidative stress resistance, and extensive studies have defined the possible pathways by which Hog1 contributes to this phenomenon (Bilsland et al., 2004). In *C. albicans*, the *Pbs2* deletion mutant exhibits a slight but reproducible increase in oxidative stress sensitivity and under such stress it loses viability faster than the *Hog1* mutant, suggesting that both Pbs2 and Hog1 have additional (and separate) roles in this stress response (Arana et al., 2005). In *V. dahliae*, Pbs2 and Hog1 played different roles in the response to H_2_O_2_, similar to the situation in *C. albicans*. The main difference between the species is that, in *V. dahliae, VdHog1* seems to be redundant rather than essential. Obviously, *VdPbs2* plays a crucial role in the response to oxidative stress.

The Δ*VdPbs2* mutant also exhibited elevated resistance to fungicides, such as iprodione and fludioxonil, which was consistent with the Δ*VdHog1* mutant in previous studies (Wang et al., 2016). Similar results were obtained in *N. crassa* (Fujimura et al., 2003; Segmuller et al., 2007) and *C. neoformans* (Kojima et al., 2006). The mechanism underlying resistance to these fungicides may involve in overstimulation of the HOG pathway (Kojima et al., 2004; Motoyama et al., 2005; Hamel et al., 2012). All mutants, as well as the wild type, were clearly sensitive to chlorothalonil, a 14α-demethylase inhibitor that acts as a broad-spectrum fungicide. However, we observed no difference in sensitivity to 2-benzo imidazole methyl carbamate and thiophanate-methyl.

In *V. dahliae*, the HOG pathway plays a significant role in fungal virulence (Tian et al., 2014; Wang et al., 2016). Here, the *VdPbs2* deletion mutant exhibited reduced virulence on smoke tree and tobacco seedlings. As we known, chitin is an essential structural component that confers rigidity to the fungal cell wall, allowing the cell to withstand chemical and physical challenges (Fesel and Zuccaro, 2015). Moreover, components of the cell wall are directly involved in colonization of host tissues and tissue damage (Lesage and Bussey, 2006; Oliveira-Garcia and Deising, 2013; Latge and Beauvais, 2014). As mentioned above, our results, *VdPbs2* mutant showed a restricted ability to penetrate into onion epidermis might be influenced by the regulation of *Pbs2* on the cell wall synthesis. Besides, the *VdPbs2* deletion mutant exhibited sensitive to H_2_O_2_, which related to ROS during host–pathogen interactions (Mehdy, 1994; Torres et al., 2005; Huang et al., 2011). Accordingly, we concluded that the changes of the cell wall synthesis and the sensitive to H_2_O_2_ in the *VdPbs2* mutants might contribute to its reduced virulence.

In summary, *VdPbs2* in *V. dahliae* is highly similar to homologs in other fungal species and acts as a key regulator during microsclerotia formation. Furthermore, deletion of *VdPbs2* has dramatic effects on cell wall synthesis, the response to stress and fungicide and virulence on smoke tree seedlings. Taken together, these results indicate that the Pbs2-Hog1 module is important for stress responses, developmental processes and pathogenicity in *V. dahliae*. Although components of the HOG MAPK signal transduction pathway in *V. dahliae, VdMsb* and *VdHog1* were recently identified and characterized, the pathway awaits further characterization, especially regarding pathogenesis. Thorough investigations may yield a clear molecular mechanism for microsclerotia formation, which could be exploited in novel approaches to disease control.

## Author Contributions

YW, CT, and LT designed the experiments. LT, JY, HZ, and DX performed the experiments and the data analyses. YW and LT prepared the figures and wrote the manuscript.

## Conflict of Interest Statement

The authors declare that the research was conducted in the absence of any commercial or financial relationships that could be construed as a potential conflict of interest.

## Footnotes

1 http://genome.jgi.doe.gov/

2 http://rsb.info.nih.gov/ij/

## Supplementary Material

FIGURE S1 **Comparison of *VdPbs2* with its homologs. (A)** Phylogenetic tree of *VdPbs2* and its homologs. The phylogenetic tree was constructed using MEGA 6.0 with full-length protein sequences. The numbers on the phylogenetic tree correspond to bootstrap values. **(B)** Amino-acid sequence alignment of *Pbs2*. Amino-acid sequence alignment of *VdPbs2* (VDAG_02783) and its homologs from *Saccharomyces cerevisiae* (YJL128C), *Neurospora crassa* (NCU00587), *Verticillium alfalfae* (VDBG_02315), *Aspergillus nidulans* (AN0931.2), *Botrytis cinerea* (XP_001553220), *Candida albicans* (XP_716629), *Ustilago maydis* (UMAG_15092), and *Magnaporthe oryzae* (MGG_00800). Conserved residues are shaded: similar residues in light gray, identical residues in dark gray. Additionally, the main conserved Pkinase domain, Pkinase_Tyr (on the sites of 322–559), is marked in the box with a dashed line.

FIGURE S2 **Disruption of *VdPbs2* in *V. dahliae*. (A)** Construction of cassette for *VdPbs2* gene disruption. The top and second lines show the two deletion cassettes, and the third line represents the open reading frame of *VdPbs2* with the 5′ and 3′ flanking regions of the wild type (XS11). The available restriction sites used for the Southern blot in this assay are marked with black arrows on the two deletion cassettes. The 1989 bp *VdPbs2* fragment (black box) was replaced by the resistance gene cassette (white box) after three homologous recombinations in the wild type. The two bottom cassettes were used for complementation and subcellular localization, respectively. P = probe. **(B)** Confirmation of gene replacement by PCR. A 2030 bp segment and no stripe were amplified in gene replacement mutants (Δ*VdPbs2-22*; Δ*VdPbs2-32*,) with external primers LY145/LY146 and internal primer pairs LY137/LY138), respectively, whereas the wild type exhibited 2788 and 327 bp bands. Using genomic DNA from the Δ*VdPbs2/Pbs2* strain as a template, bands at 2030 bp and 327 bp were amplified using primer pair LY105/LY166. **(C)** Validation of gene replacement in the two *VdPbs2* deletion mutants by Southern blotting. The 527 bp band demonstrates that the Δ*VdPbs2-22* is a single-copy knockout. Δ*VdPbs2-32* was a two-copy knockout (data not shown).

FIGURE S3 **The phenotypic assays of Δ*VdPbs2/Pbs2 GFP* strain. (A)** Δ*VdPbs2/Pbs2GFP* strain restores the reduced microsclerotia formation on BM plates. **(B)**Δ*VdPbs2/Pbs2GFP* recovers the fungal growth under osmotic and cell wall inhibitor agents, respectively. **(C)** The growth rate of Δ*VdPbs2/Pbs2GFP* strain. These entire assays were performed in triplicate and same as the phenotype analysis of Δ*VdPbs2* strains.

FIGURE S4 **Penetration of the *VdPbs2* deletion strain into onion epidermis. (A)** Penetration assays on onion epidermis revealed restricted penetration by Δ*VdPbs2*. Inoculations were performed with 10^4^/ml conidia. Images were acquired at 36 hpi. **(B)** The percentage of penetration. The penetration of the Δ*VdPbs2* and the wild type were performed after at 36 hpi.

TABLE S1 **PCR primers used in this study**.
