# Supplementary figures and images for: The Mitogen-Activated Protein Kinase Kinase VdPbs2 of Verticillium dahliae Regulates Microsclerotia Formation, Stress Response, and Plant Infection
Source: Front Microbiol. 2016 Sep 27;7:1532. doi: 10.3389/fmicb.2016.01532 (PMC5037172; doi:10.3389/fmicb.2016.01532)

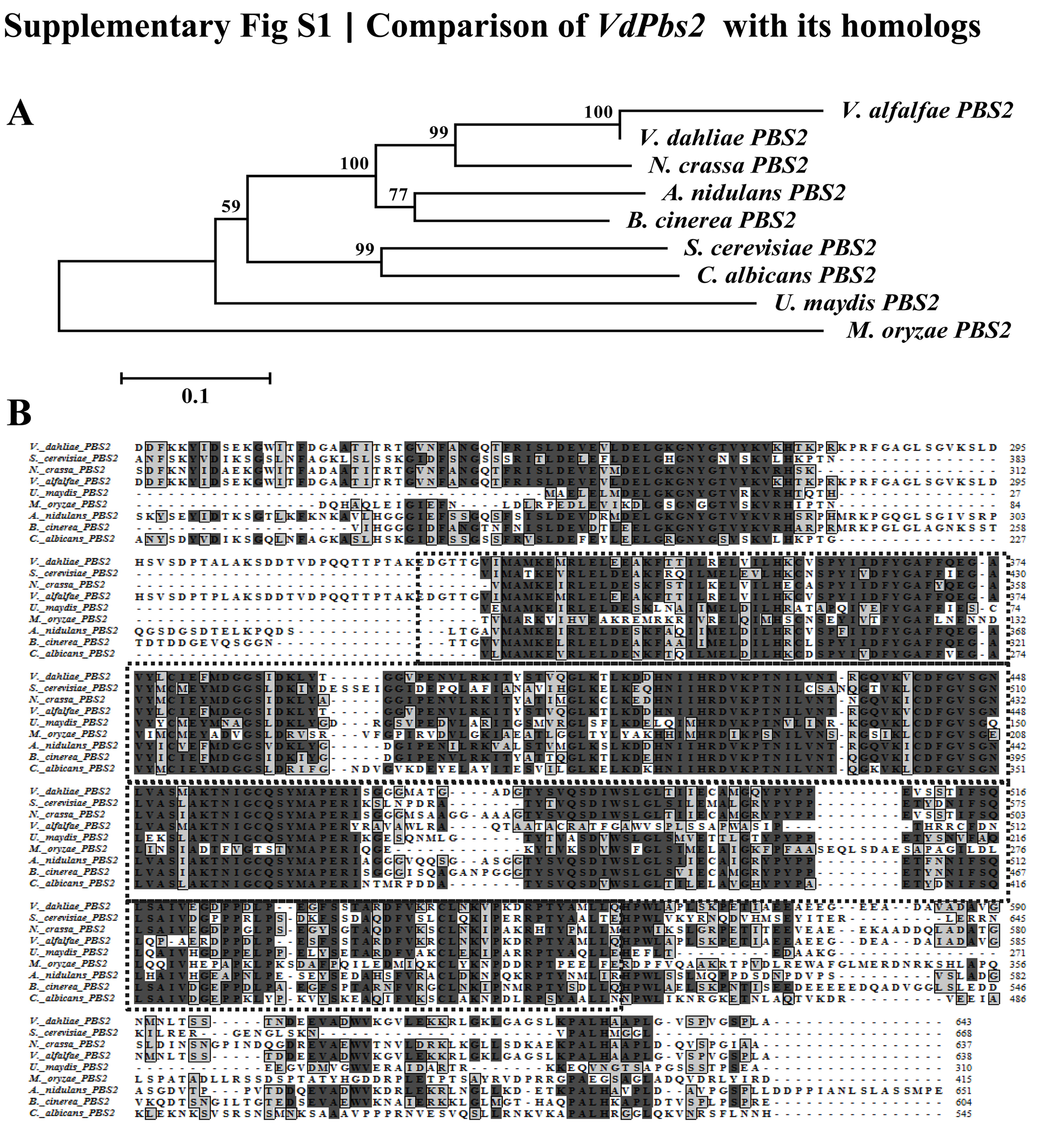

Supplement: FIGURE S1 — Comparison of VdPbs2 with its homologs. (A) Phylogenetic tree of VdPbs2 and its homologs. The phylogenetic tree was constructed using MEGA 6.0 with full-length protein sequences. The numbers on the phylogenetic tree correspond to bootstrap values. (B) Amino-acid sequence alignment of Pbs2. Amino-acid sequence alignment of VdPbs2 (VDAG_02783) and its homologs from Saccharomyces cerevisiae (YJL128C), Neurospora crassa (NCU00587), Verticillium alfalfae (VDBG_02315), Aspergillus nidulans (AN0931.2), Botrytis cinerea (XP_001553220), Candida albicans (XP_716629), Ustilago maydis (UMAG_15092), and Magnaporthe oryzae (MGG_00800). Conserved residues are shaded: similar residues in light gray, identical residues in dark gray. Additionally, the main conserved Pkinase domain, Pkinase_Tyr (on the sites of 322–559), is marked in the box with a dashed line. [file Image_1.JPEG]

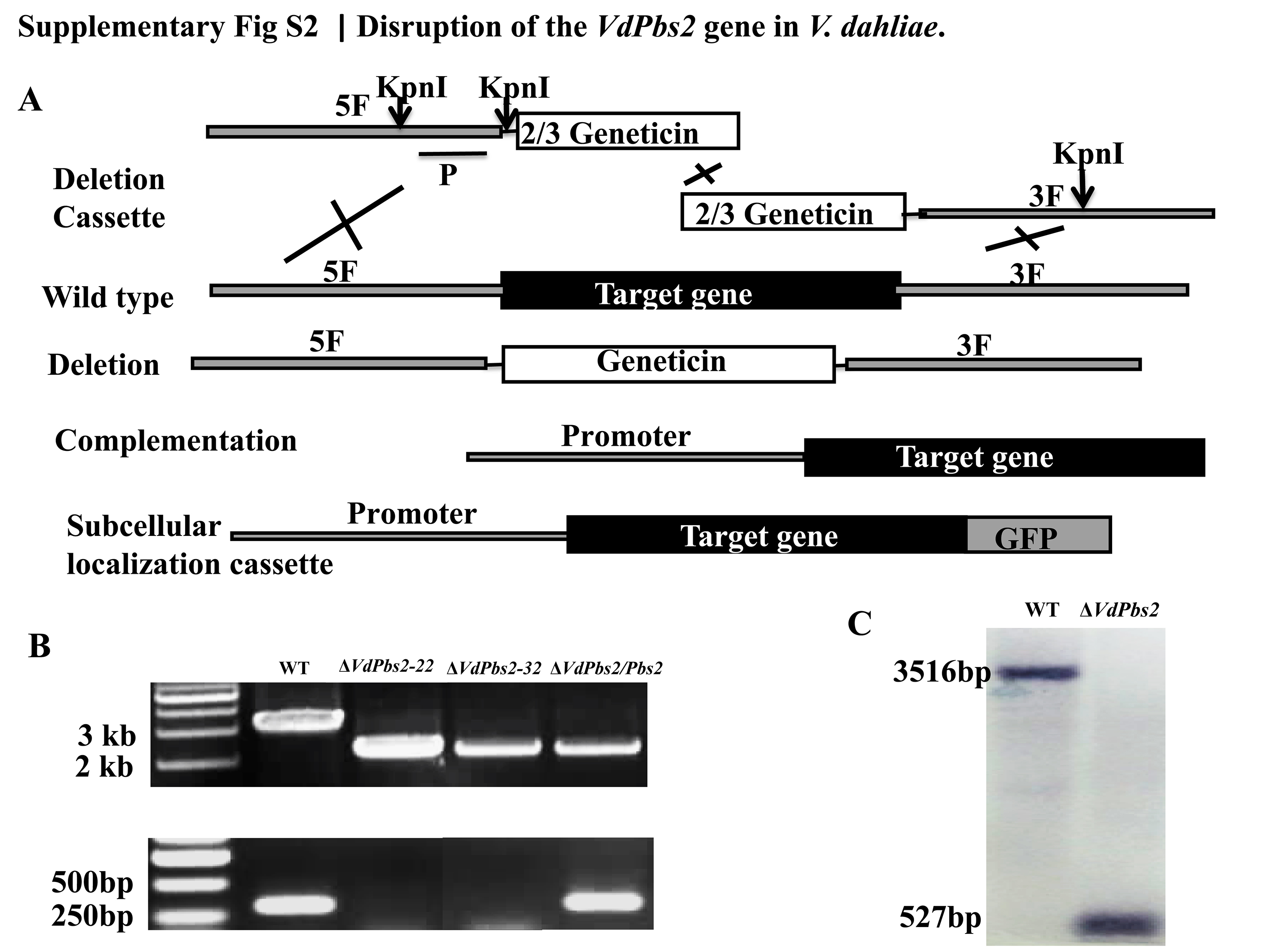

Supplement: FIGURE S2 — Disruption of VdPbs2 in V. dahliae. (A) Construction of cassette for VdPbs2 gene disruption. The top and second lines show the two deletion cassettes, and the third line represents the open reading frame of VdPbs2 with the 5′ and 3′ flanking regions of the wild type (XS11). The available restriction sites used for the Southern blot in this assay are marked with black arrows on the two deletion cassettes. The 1989 bp VdPbs2 fragment (black box) was replaced by the resistance gene cassette (white box) after three homologous recombinations in the wild type. The two bottom cassettes were used for complementation and subcellular localization, respectively. P = probe. (B) Confirmation of gene replacement by PCR. A 2030 bp segment and no stripe were amplified in gene replacement mutants (ΔVdPbs2-22; ΔVdPbs2-32,) with external primers LY145/LY146 and internal primer pairs LY137/LY138), respectively, whereas the wild type exhibited 2788 and 327 bp bands. Using genomic DNA from the ΔVdPbs2/Pbs2 strain as a template, bands at 2030 bp and 327 bp were amplified using primer pair LY105/LY166. (C) Validation of gene replacement in the two VdPbs2 deletion mutants by Southern blotting. The 527 bp band demonstrates that the ΔVdPbs2-22 is a single-copy knockout. ΔVdPbs2-32 was a two-copy knockout (data not shown). [file Image_2.JPEG]

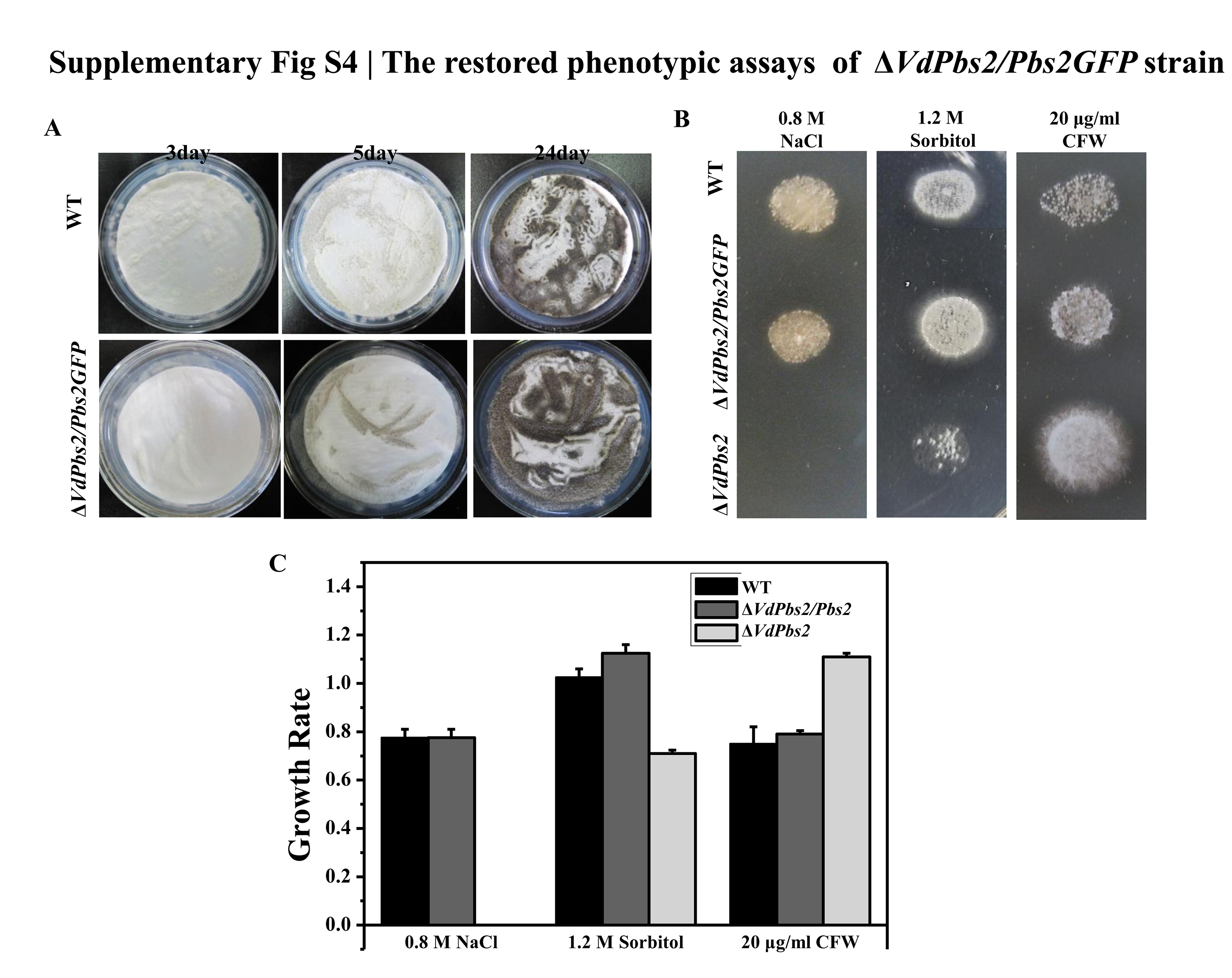

Supplement: FIGURE S3 — The phenotypic assays of ΔVdPbs2/Pbs2 GFP strain. (A) ΔVdPbs2/Pbs2GFP strain restores the reduced microsclerotia formation on BM plates. (B)ΔVdPbs2/Pbs2GFP recovers the fungal growth under osmotic and cell wall inhibitor agents, respectively. (C) The growth rate of ΔVdPbs2/Pbs2GFP strain. These entire assays were performed in triplicate and same as the phenotype analysis of ΔVdPbs2 strains. [file Image_3.JPEG]

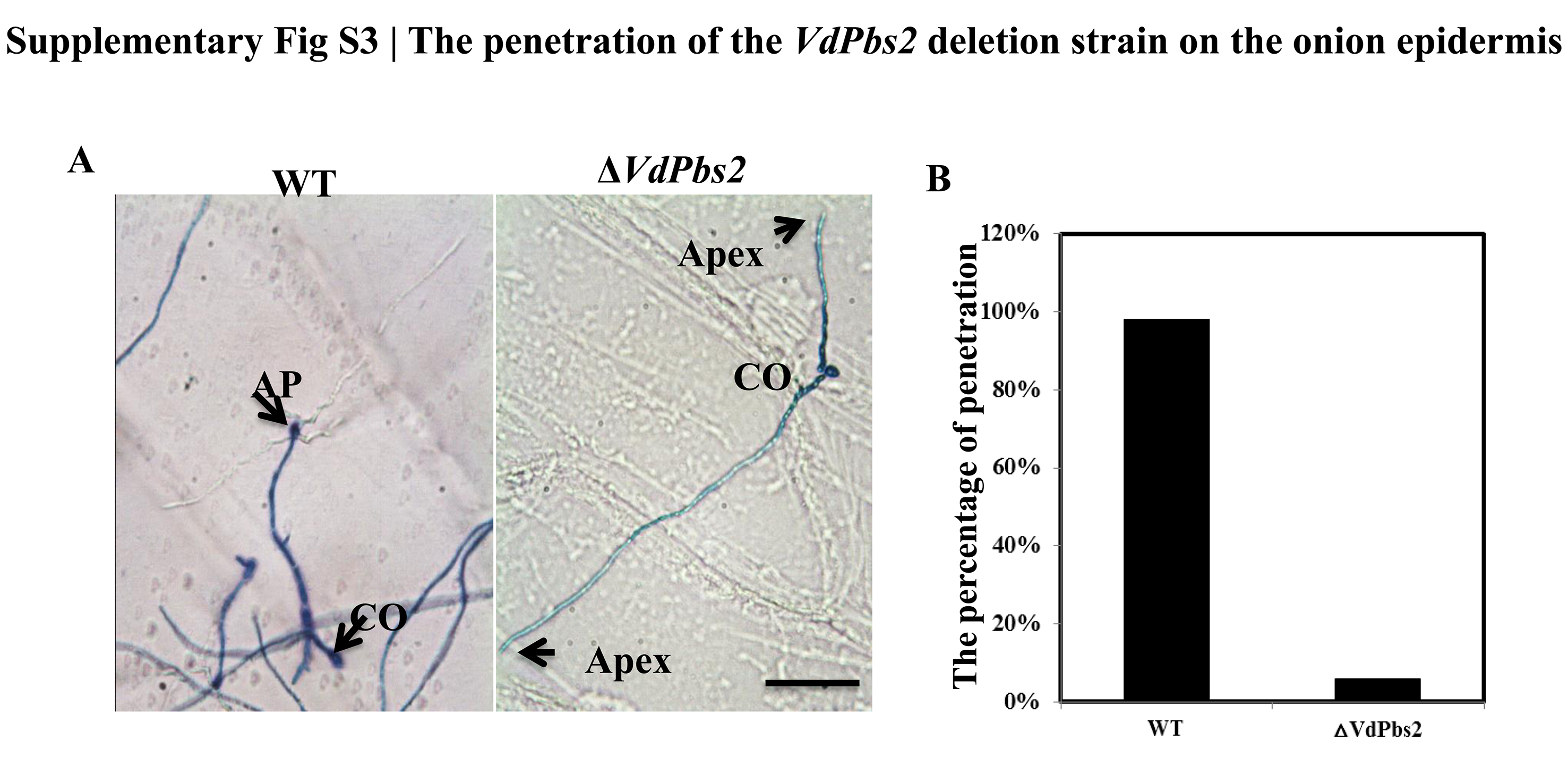

Supplement: FIGURE S4 — Penetration of the VdPbs2 deletion strain into onion epidermis. (A) Penetration assays on onion epidermis revealed restricted penetration by ΔVdPbs2. Inoculations were performed with 104/ml conidia. Images were acquired at 36 hpi. (B) The percentage of penetration. The penetration of the ΔVdPbs2 and the wild type were performed after at 36 hpi. [file Image_4.JPEG]
