# Supplementary material for: The Mitogen-Activated Protein Kinase Kinase VdPbs2 of Verticillium dahliae Regulates Microsclerotia Formation, Stress Response, and Plant Infection
Source: Front Microbiol. 2016 Sep 27;7:1532. doi: 10.3389/fmicb.2016.01532 (PMC5037172; doi:10.3389/fmicb.2016.01532)
Supplement: TABLE S1 — PCR primers used in this study. [file Table_1.DOCX]

**Supplementary Table 1 PCR primers used in this study**

| Primer name | Description | Primer sequence (5’–3’) |
| --- | --- | --- |
| LY105 | Vdpbs2 5’ end deletion | CTTCCCTCCTTGGCTCTT |
| LY106 |  | gtcgtgactgggaaaaccctggcgTTATACTGCACGCTTGTCG |
| LY107 | Vdpbs2 3’ end deletion | tcctgtgtgaaattgttatccgctCATAGCATCGGGTGAATACG |
| LY108 |  | CTTCTGCGGTGACAACAA |
| LY137  LY138 | Internal screening primer | CCAGGTCAAGGTCTGCGATT |
|  |  | GGTGGGTCACCTTCGACAAT |
| LY145  LY146 | External screening primer | TGCTCCACGAACACTTC |
|  |  | CACAGCTCAACCACCTC |
| PL5 | HPH resistance cassette | GGAGGTCAACACATCAATGCCT |
| PL131 |  | CCTCGGACGAGTGCTGGGGCGT |
| Geneticinfor | Geneticin cassette | CGCCAGGGTTTTCCCAGTCACGAC |
| Geneticinrev |  | AGCGGATAACAATTTCACACAGGA |
| LY166 | Construction for complementation | TGCTGCAATATCGATGGCTT |
| LY167 | Construction of Vdpbs2::eGFP | GCCGACGGGACTGACACCGA |
| LY165-RB | Screening primer of VdPBS2::eGFP construct | ACGCTCTTTTCTCTTAGGTTT |
| VdBt-up | Amplification of β-tubulin | AgCTCACCCAgCAgATgTTC |
| VdBt-down |  | TCgACCTCCTTCATggCAAC |
| VDAG_08591 F | qRT-PCR of VDAG_08591 homologs of CHS2 | TTGTTCTGGCTCTGGGTAA |
| VDAG_08591 R |  | GATTCGAGGCTGATTTGG |
| VDAG_03141 F | qRT-PCR of VDAG_03141 homologs of CHS4 | TCATGGCGAAGAAATGGC |
| VDAG_03141 R |  | CGTAGAAACCGCAGTAGACC |
| LY170-up | Probe for hybridization | ACCTACCTAGGCAAGCTAGGT |
| LY170-down |  | TTATACTGCACGCTTGTCG |
| PW21 | qRT-PCR for VDAG_03674 | CAAGTCGATCGGCATGAAGG |
| PW22 |  | CACCAACCCCATCTCTCACT |
| PW25 | qRT-PCR for VDAG_00190 | CTCGTCCTGATCCGTATCCCA |
| PW26 |  | TGTGAATTGAGGCAGGCATG |
| PW29 | qRT-PCR for VDAG_03665 | TGGCATCAAGACAGACATGTA |
| PW30 |  | ACAACGCGAGCGATGTCGAT |
| PW33 | qRT-PCR for VDAG_03393 | AAAGGTGTTTGAGAGCGGAC |
| PW34 |  | ATCTCCCTCTCCACAACAGC |
| PW37 | qRT-PCR for VDAG_00183 | TATGTCCCTGGCGGCTTTAA |
| PW38 |  | TGATCCACTCGCAGTCTTCA |
| LY204 | qRT-PCR for VDAG_06340 | CGTCACAGCGGAGCAGTAC |
| LY205 |  | GCACATTGATGGCGAGCAT |
| LY212 | qRT-PCR for VDAG_08724 | CTTCACTTCAGCAGCAACCTC |
| LY213 |  | CAGTAGGCACGGGAACGAG |
| LY214 | qRT-PCR for VDAG_03661 | TTCCAACAGCACCAGCAA |
| LY215 |  | GCAATACGGAGCCAATCA |
| LY218 | qRT-PCR for VDAG_02630 | CGCTCCTCCTTACCTTGGG |
| LY219 |  | GCGTCGGTCTTGATGTTGC |
